# Frailty and Associated Risk Factors in Elderly People with Health Examination in Rural Areas of China

**Published:** 2019-09

**Authors:** Jie GU, Haiying CHEN, Xiaoqing GU, Xiaomin SUN, Zhigang PAN, Shanzhu ZHU, Doris YOUNG

**Affiliations:** 1. Zhongshan Hospital of Fudan University, Shanghai 200032, P.R. China; 2. Xidu Community Health Center of the Fengxian District, Shanghai 201400, P.R. China; 3. Health Development Institute of Pudong, Shanghai 201208, P.R. China; 4. Department of General Practice, Melbourne Medical School, The University of Melbourne, Melbourne, Australia

**Keywords:** Rural, Elderly people, Fried frailty phenotype definition, Frailty

## Abstract

**Background:**

Frailty is a common syndrome in elderly people, but has not been fully studied in China. We aimed to investigate the epidemiological characteristics of frailty and analyze its risk factors in elderly people in rural areas of China.

**Methods:**

This cross-sectional study was conducted between September and October 2016. Overall, 4323 elderly people over 60 yr were enrolled by cluster random sampling method from the Fengxian District of Shanghai, China. These subjects voluntarily participated in the health examination of the 2016 National Basic Public Health Service Program. In addition to regular examination items, frailty assessment was performed on the basis of Fried frailty phenotype criteria.

**Results:**

The prevalence of pre-frailty and frailty was 49.4% and 6.8%, respectively. Advanced age (OR=1.44 for pre-frailty and OR=2.01 for frailty, 65–74 years old; OR=3.02 for pre-frailty and OR=14.13 for frailty,75–84 years old; OR=8.17 for pre-frailty and OR=71.71 for frailty, ≥85 years old), female (OR=1.32 for pre-frailty and OR=1.97 for frailty), stroke history (OR=1.75 for pre-frailty and OR=2.43 for frailty), vision decrease (OR=1.98 for pre-frailty and OR=2.70 for frailty), and anemia (OR=1.95 for pre-frailty and OR=3.64 for frailty) were common risk factors for both pre-frailty and frailty.

**Conclusion:**

Healthy elderly people in the rural areas of Shanghai have relatively high prevalence of pre-frailty and frailty. Advanced age, female, stroke history, decreased vision, and anemia are the risk factors for pre-frailty and frailty.

## Introduction

Frailty, a common clinical syndrome in elderly people, gains increasing attention in recent years (1). The significance of frailty lies in the accelerated decrease of body’s physiological function and loss of the ability to maintain a stable state, leading to the increase of the risk of fall, mental disorder, disability, hospitalization, and even death (2,3).

Two conventional methods used in the assessment of frailty include frailty phenotype definition and accumulated deficits model (4). Fried et al. defined frailty phenotype criteria, that is, ≥3 items in the following 5 items: 1) unintentional weight loss; 2) slow walking speed; 3) weakness; 4) fatigue; 5) low physical activity. Mitnitski et al. developed frailty index (FI) which calculates accumulated deficits of a variety of indexes such as symptoms, signs, lab test abnormalities, functional impairments, etc. (5).

Frailty research has just begun in China. Although scholars recognize FFP as currently the most widely used method worldwide (6), most of studies in China use FI to assess frailty (7–10). The large variation in the content and quantity of FI indicators in different studies often leads to the loss of comparability between studies. On the other hand, there were also drawbacks of a few of studies in which FFP was used; for example, to determine the presence of weakness according to self-reported “difficult to lift or transport 10 kg object” instead of grip force measurement (11). Therefore, the standardization of FFP to assess the frailty state of the Chinese population is of practical significance.

In recent years, components of the “National Basic Public Health Service Program” in China have been increasing (12). Free annual health examination for the elderly, one component of the program, exerts an important role in the disease prevention and health management, and also provides a platform for gerontological studies. In addition, the urban-rural gap is the main problem in the distribution of medical resources in China (13). There are a large number of elderly people in rural areas of China who are the key populations to achieve equalization of basic public health services (14).

However, regarding the frailty research in China, there is no study targeting them so far. Therefore, in this study, FFP method was used to carry out an epidemiological investigation of frailty and analyze potential risk factors.

## Materials and Methods

### Subjects

#### Basic information of the rural areas in Shanghai and Fengxian District

By the end of 2014, Shanghai had a total of 1,593 administrative villages and an agricultural population of 1,319,900. Fengxian District is located in the south of Shanghai, which is a rural area with relatively backward economic development (15). The report of Shanghai Municipal Bureau of Statistics showed, the GDP of this district accounted for 2.7% of the total GDP of Shanghai, and ranked 15th in the 16 municipal districts of Shanghai in 2014.

#### Sampling method

Health examination for elderly people was implemented by the Community Health Center (CHC) in Shanghai. There are 22 CHCs in Fengxian District. Cluster random sampling with the CHC as sampling unit was used. In this study, the Xidu CHC was randomly selected from Fengxian District by random number table method. There are 10 administrative villages in this community.

#### Inclusion and exclusion criteria

Inclusion criteria: 1) Residents of the community who participated in the health examination in the Xidu CHC; (2) ≥ 60 yr; 3) registered household address and actual residence address are both in the sampled villages; 4) voluntary acceptance of frailty assessment. Exclusion criteria: 1) Unable to complete survey because of hearing loss or other reasons; 2) unable to complete grip strength or walking speed measurement because of fracture, trauma or other reasons.

## Methods

### Study design

This was a cross-sectional study. Frailty assessment was carried out based on FFP method when study subjects participated in the health examination in the Xidu CHC from September to October 2016.

### Items of the health examination

Items of the health examination were included by the National Health and Family Planning Commission, including 4 parts, lifestyle and health status assessment questionnaire survey, physical examination, laboratory examination and health guidance (16). Items used in the present study included: 1) Chronic diseases: including hypertension, diabetes, coronary heart disease (CHD), chronic obstructive pulmonary disease (COPD), stroke, chronic kidney disease (CKD) and cancer. Data were obtained by self-reported medical history. Diabetes and CKD were also diagnosed if fasting plasma glucose (FPG) ≥7.0 mmol/L or eGFR <60 mL/(min • 1.73m^2^), correspondingly. 2) Physical exercise: including 4 choices, no, occasionally, weekly and daily exercise. 3) Body Mass Index (BMI): BMI was calculated automatically by the fully automatic height and weight scale (WS-H16, Shanghai Woshen). Obesity was defined as BMI ≥28 kg/m^2^. 4) Hearing: “clearly hearing” and “not clearly hearing or inaudible”, judged by examiners using voice test. Subjects choosing the latter were defined as hearing loss. 5) Vision: standard logarithmic visual acuity chart was used. According to the WHO 1973 standard, visual acuity <4.5 was defined as vision decrease. 6) Activity of Daily Living (ADL) assessment: 6 basic ADLs and 8 instrumental ADLs put forward by Lawton and Brody were used (17). Each item had 4 choices: fully complete by self, a little difficult, need assistance and unable to complete, counting 1, 2, 3, and 4 points. Disability was defined as total score >16 points. 7) Laboratory test: Automatic blood analyzer (XS-900i, Sysmex Corporation) was used for hemoglobin (Hb) test. Anemia was defined as male Hb<120 g/L, female Hb<110 g/L according to the Chinese standard. Biochemical indexes were tested by automatic biochemical analyzer (Accute TBA-40FR, Toshiba), including urea nitrogen, creatinine, total cholesterol (TC), triglyceride (TG), FPG. eGFR was calculated using the MDRD formula.

### Frailty assessment

According to FFP, among the following five conditions, those without any one of them were healthy, those with 1–2 conditions were pre-frailty, and those with ≥ 3 conditions were frailty. 1) Unintentional weight loss: defined as body weight decreased by ≥ 4.5 kg or 5% compared with one year ago, without any special methods such as diet, drugs, etc. 2) decrease of walking speed: Examinees were required to walk 15 ft (4.57 m) at their usual walking speed, and stopwatch was used for recording time. Decrease of walking speed was defined as walking speed <0.8 m/s (4, 18), or needing auxiliary walking equipment or human assistance while walking. 3) Weakness: if the maximum value of grip strength <26 kg in male or <18 kg in female, it was defined as weakness. Digital dynamometer (Jamar Plus, JAMAR) was used to measure grip strength of both hands. 4) Fatigue: defined as “Yes” when being asked “whether do you often feel fatigue?”. 5) Low physical activity: defined as “low group” in the short form of international physical activity questionnaire (IPAQ-SF) being used for assessing physical activity in this study (19).

### Quality control

There were 21 investigators and 8 frailty assessors, all of whom were general practitioners of the Xidu CHC. Investigators were responsible for questionnaire survey and physical examinations in the health examination section, receiving intensive training focused on the explanation of the contents of the questionnaire. Frailty assessors were trained for the use of dynamometer and the measurement of walking speed. Age, gender, and FFP’s five diagnostic conditions were core data. If any part of the core data were missing, the corresponding subject was excluded.

### Ethical Approval

All procedures performed in the study involving human participants were in accordance with the ethical standards of the institutional and/or national research committee and with the 1964 Helsinki declaration and its later amendments or comparable ethical standards.

### Statistical analysis

Data analyses were performed using SPSS 22.0 software. Normal distribution data were compared by *t* test or ANOVA. Categorical variables were compared using chi-square test or trend chi-square test. Logistic regression was used for risk factor analysis. *P* < 0.01 was considered statistically significant.

## Results

### General information and frailty prevalence

A total of 5166 elderly people participated in the health examination of the Xidu CHC in 2016. Overall, 685 people were excluded according to the exclusion criteria, and 158 people were excluded due to incomplete core data. At last, 4323 people were included, accounting for 83.7% (4323/5166) of the people undergoing health examination. The average age of the included subjects was 70.2±7.0 yrs, and male accounted for 41.5% (1792/4323).

The prevalence of pre-frailty and frailty was 49.4% (2135/4323) and 6.8% (294/4323). Among the three groups of healthy, pre-frailty and frailty, except hypertension, diabetes, COPD, cancer, obesity, TC and TG, all other conditions or indexes showed significant differences (Table 1).

**Table 1:** Comparison of general conditions of healthy, pre-frailty and frailty subjects

***Variable***	***Total (n=4323)***	***Frailty status***				
		***Healthy (n=1894)***	***Pre-frailty (n=2135)***	***Frailty (n=294)***	***t / χ^2^***	***P***
Age (yr, x̄±s)	70.2±7.0	67.9±5.5	71.2±7.2	78.2±7.3	362.092^a^	<0.001
Male (%)	1792(41.5)	884(46.7)	824(38.6)	84(28.6)	48.588^b^	<0.001
Chronic disease (%)												
Hypertension	2750(63.6)	1172(61.9)	1375(64.4)	203(69.0)	6.785^b^	0.034
Diabetes	480(11.1)	203(10.7)	230(10.8)	47(16.0)	7.623^b^	0.022
CHD	111(2.6)	43(2.3)	67(3.1)	1(0.3)	9.278^b^	0.010
COPD	102(2.4)	33(1.7)	60(2.8)	9(3.1)	5.643^b^	0.060
Stroke	273(6.3)	72(3.8)	164(7.7)	37(12.6)	46.500^b^	<0.001
CKD	116(2.7)	26(1.4)	68(3.2)	22(7.5)	40.452^b^	<0.001
Cancer	26(0.6)	8(0.4)	17(0.8)	1(0.3)	2.707^b^	0.258
Physical exercise (%) (n=4303)												
No	3253(75.6)	1355(72.0)	1647(77.4)	251(85.7)	33.317^b^	<0.001
Occasionally	93(2.2)	47(2.5)	43(2.0)	3(1.0)		
Weekly	86(2.0)	44(2.3)	37(1.7)	5(1.7)		
Daily	871(20.2)	435(23.1)	402(18.9)	34(11.6)		
BMI(kg/m^2^, x̄±s)	23.8±3.3	24.1±3.1	23.7±3.4	22.6±3.6	33.188^a^	<0.001
Obesity (%)	426(9.9)	196(10.3)	211(9.9)	19(6.5)	4.330^b^	0.115
Hearing loss (%) (n=4026)	115(2.9)	26(1.5)	69(3.5)	20(7.1)	32.886^b^	<0.001
Vision decrease (%) (n=4043)	345(8.5)	72(4.0)	211(10.6)	62(23.9)	10.169^b^	0.001
Disability (%) (n=4163)	114(2.1)	15(0.8)	63(3.0)	36(12.4)	127.353^b^	<0.001
Anemia (%) (n=4227)	124(2.9)	20(1.1)	76(3.7)	28(9.7)	72.890^b^	<0.001
TC (mmol/L, x̄±s) (n=4227)	4.98±0.90	5.00±0.89	4.97±0.91	4.94±0.91	0.688^a^	0.503
TG (mmol/L, x̄±s) (n=4227)	1.60±1.06	1.63±1.10	1.58±1.05	1.51±0.79	2.034^a^	0.131

Note:

a represents *t* value of *t* test;

b represents χ*^2^* value of chi-square test.

Stratified by gender, the prevalence of pre-frailty and frailty was 46.0% (824/1792) and 4.7% (84/1792) in male, and 51.8% (1311/2531) and 8.3% (210/2531) in female. Female had significantly higher prevalence of both pre-frailty and frailty than male (χ^2^=14.196, *P*<0.001 for pre-frailty; χ^2^=21.567, *P*<0.001 for frailty).

In the group of advanced age (≥ 85 yr), the prevalence of pre-frailty and frailty was 57.8% (107/185) and 33.5% (62/185); however, male and female had no significant differences (pre-frailty:55.4% vs. 59.5%, χ*^2^*=0.29, *P*=0.584; frailty: 33.8% vs. 33.3%, χ*^2^*=0.004, *P*=0.949) (Table 2).

**Table 2:** Correlation between frailty and age and gender

***Variable***	***Healthy (n=1894)***	***Pre-frailty (n=2135)***	***Frailty (n=294)***	***χ^2^***	***P***
Male (%)					
60–64	280(61.1)	174(38.0)	4(0.9)	174.450	<0.001
65–74	486(53.5)	406(44.7)	17(1.9)		
75–84	110(31.3)	203(57.8)	38(10.8)		
≥85	8(10.8)	41(55.4)	25(33.8)		
Total	884(49.3)	824(46.0)	84(4.7)		
Female (%)					
60–64	349(55.5)	267(42.4)	13(2.1)	331.929	<0.001
65–74	557(44.6)	643(51.5)	49(3.9)		
75–84	96(17.7)	335(61.8)	111(20.5)		
≥85	8(7.2)	66(59.5)	37(33.3)		
Total	1010(39.9)	1311(51.8)	210(8.3)		
Total (%)					
60–64	629(57.9)	441(40.6)	17(1.6)	506.301	<0.001
65–74	1043(48.3)	1049(48.6)	66(3.1)		
75–84	206(23.1)	538(60.2)	149(16.7)		
≥85	16(8.6)	107(57.8)	62(33.5)		
Total	1894(43.8)	2135(49.4)	194(6.8)		

### Trend analysis of frailty and age

The subjects were divided into four age groups. Trend chi-square test showed no matter in males (*χ^2^*=174.450, *P*<0.001), females (*χ^2^*=331.929, *P*<0.001) or the whole population (*χ^2^*=506.301, *P*<0.001), frailty degree increased with increasing age (Table 2).

### Multivariate logistic regression analysis of risk factors for pre-frailty and frailty

The frailty status was dependent variable (“healthy” was the reference), and potential risk factors were independent variables. Model 1 was univariate analysis. In model 2, significant factors identified in the model 1 (*P* < 0.05) were included for multivariate analysis. The results showed that age, female, stroke history, vision decrease, anemia were common risk factors for pre-frailty and frailty; disability were risk factors for frailty; daily physical exercise was a common protection factor for pre-frailty and frailty (Table 3).

**Table 3:** Risk factor analysis for pre-frailty and frailty *vs.* healthy

***Variable***	***Model 1***				***Model 2***			
	***Pre-frailty***		***Frailty***		***Pre-frailty***		***Frailty***	
	***OR***	***P***	***OR***	***P***	***OR***	***P***	***OR***	***P***
Age range (*vs.* 60–64 yr)								
65–74	1.44	<0.001	2.34	0.002	1.44	<0.001	2.01	0.004
75–84	3.73	<0.001	26.76	<0.001	3.02	<0.001	14.13	<0.001
≥85	9.54	<0.001	143.38	<0.001	8.17	<0.001	71.71	<0.001
Female	1.39	<0.001	2.39	<0.001	1.31	0.006	1.97	0.003
Chronic diseases								
Hypertension	1.12	0.097	1.37	0.018	0.99	0.912	1.06	0.782
Diabetes	1.01	0.955	1.59	0.009	0.73	0.217	0.75	0.630
CHD	1.40	0.093	0.15	0.058	Not included			
COPD	1.63	0.026	1.78	0.130	1.54	0.107	1.01	0.981
Stroke	2.11	<0.001	3.64	<0.001	1.75	0.005	2.43	0.008
CKD	2.36	<0.001	5.81	<0.001	1.50	0.595	4.53	0.153
Cancer	1.89	0.138	0.81	0.838	Not included								
Physical exercise (*vs.* No)								
Occasionally	0.75	0.103	0.35	0.076	0.73	0.215	0.29	0.116
Weekly	0.69	0.185	0.61	0.306	0.77	0.316	1.30	0.619
Daily	0.76	<0.001	0.42	<0.001	0.76	0.003	0.42	0.001
Obesity	0.95	0.625	0.60	0.039	0.97	0.790	0.87	0.625
Hearing loss	2.40	<0.001	5.07	<0.001	1.63	0.067	1.30	0.517
Vision decrease	2.82	<0.001	7.50	<0.001	1.98	<0.001	2.70	<0.001
Disability	3.75	<0.001	16.92	<0.001	1.43	0.277	2.97	0.007
Anemia	3.48	<0.001	9.90	<0.001	1.95	0.019	3.61	0.001
TC≥6.2 mmol/L	1.02	0.861	0.81	0.389	Not included			
TG≥2.3 mmol/L	0.88	0.147	0.65	0.036	0.90	0.336	0.78	0.323

## Discussion

Health examination for elderly people is a basic public health service in China. Unlike the elderly who are hospitalized, the elderly participating in health examination come from communities and are the best representatives of general elderly people. In Chinese rural areas where medical resources are relatively insufficient, health examination is of greater significance for the early detection of diseases and maintenance of health in the elderly (20). Therefore, the present study chose rural elderly people receiving health examination as study subjects to explore the frailty status in this population.

There are several studies using FFP in China. For example, Zhu et al. (11) found the prevalence of frailty in the elderly aged 70–84 was 11.3%. In Taiwan (21), the prevalence in the elderly over 50 was 6.8%. In addition, the study in Hong Kong, the elderly over 60 has the prevalence of 6.2% (22).

In our study, the prevalence of frailty was 6.8% in the subjects over 60 years old, and soared to more than 30% in the subjects over 85 years old. Although the characteristics of the study subjects in these studies are different, they obtained similar results with our study that Chinese elderly people have relatively high prevalence of frailty (at least 6%). Furthermore, the systematic review of Collard et al (6) showed, based on FFP assessment, the prevalence of frailty in elderly people aged 65 or above was 9.9%; while the combined data showed female had 9.6% prevalence, which was higher than male (5.2%). Our results were consistent with results obtained in other countries.

We also found that regardless of gender, the prevalence of frailty increased with age, which has been confirmed by previous studies (6). In fact, in our study, advanced age was the most significant risk factor for frailty. After adjusting other factors, compared with 60–64 age-range group, the risks in other three age-range groups, 65–74, 75–84, and ≥85 to have frailty were 2.01 times, 14.13 times and 71.71 times, and 1.44 times, 3.02 times and 8.17 times for pre-frailty, correspondingly, reminding us that the assessment and management of frailty in elderly people should receive sufficient attention.

Besides advanced age, female, stroke history, decreased vision and anemia were also common risk factors for frailty and pre-frailty. The findings were in line with the findings of Ng et al. (23). Effect of gender on frailty had been discussed. As to stroke, patients with a stroke history often have sequelae including certain physical dysfunctions (24), leading to weakness, slowness and low physical activity, which are the key components of FFP criteria. In recent years, research on stroke-related sarcopenia further clear the causal relationship between stroke and frailty (25). Decreased vision can seriously affect the daily life of patients, leading to a decline in mental health, autonomy and mobility (26). Anemia patients often have fatigue, and anemia in elderly is often caused by chronic inflammation (27); while inflammation is a likely reason of frailty (28). Daily physical exercise is a common protective factor for pre-frailty and frailty; in fact, exercise intervention is one of the means to treat frailty (29).

An interesting finding in the univariate logistic analysis in this study was that obesity and TG≥2.3 mmol/L were almost statistically protective factors for frailty although the two factors became not significant in the multivariate analysis. Many studies have shown malnutrition and low-weight are risk factors for frailty (30, 31), while obesity and hypertriglyceridemia usually suggest the presence of overnutrition. However, the study of Garcia-Esquinas et al. suggested that both ordinary obesity and abdominal obesity increased the risk of frailty in elderly (32), and Hubbard et al. found that BMI and frailty had a U-shaped correlation, which means people with too low or too high BMI were prone to frailty (33). Therefore, the relationship between obesity and frailty still needs studies to demonstrate.

### Limitation

This study had two limitations: (1) Although the sample size was large and subjects came from 10 administrative villages, all subjects were covered by one CHC, which was geographically concentrated and may affect the representativeness of the samples; (2) limited by the health examination items, the subjects did not receive the evaluation of cognitive function and emotional state.

## Conclusion

Pre-frailty and frailty have high prevalence among elderly people receiving health examination in the rural areas of Shanghai. Elderly females who have stroke history, decreased vision and anemia should be given special attention since they are high-risk population for pre-frailty and frailty.

## Ethical considerations

Ethical issues (Including plagiarism, informed consent, misconduct, data fabrication and/or falsification, double publication and/or submission, redundancy, etc.) have been completely observed by the authors.
